# Preventing Adipogenesis and Preserving Mitochondria and GLUT-4 Functions by Extracts and Isolated Compounds of Australian *Acacia saligna*

**DOI:** 10.3390/molecules28186677

**Published:** 2023-09-18

**Authors:** Anjar P. Asmara, Hui Chen, Alison T. Ung

**Affiliations:** 1 School of Mathematical and Physical Sciences, Faculty of Science, University of Technology Sydney, Ultimo, NSW 2007, Australia; anjarpurba.asmara@student.uts.edu.au; 2 School of Life Sciences, Faculty of Science, University of Technology Sydney, Ultimo, NSW 2007, Australia; hui.chen-1@uts.edu.au

**Keywords:** *Acacia saligna*, antiadipogenic, type 2 diabetes mellitus, mitochondrial biogenesis, naringenin-*7*-*O*-*α*-*L*-arabinofuranoside, (−)-epicatechin, *D*-(+)-pinitol, GLUT-4

## Abstract

*Acacia saligna*’s secondary metabolites show promise in treating type 2 diabetes mellitus and its related conditions. We previously discovered that methanolic extracts, isolated flavonoids, and cyclitols effectively preserve mitochondria in 3T3-L1 adipocytes. In this current work, quantification of lipid droplet levels with Oil Red O assay showed a noticeable decrease in lipogenesis in 3T3-L1 cells. Methanolic leaf and bark extracts and isolated compounds, (−)-epicatechin **6** and myricitrin **8**, reduced cellular lipid levels by 21.15% to 25.28%, respectively. mRNA levels of key regulators of mitochondrial biogenesis, such as adiponectin, PGC-1α, and mtTFA, were increased. Methanolic flower extract (FL-MeOH) and its chemical components, naringenin **1** and *D*-(+)-pinitol **5a**, increased these gene levels from 10% to 29% at the higher dose. Our study found that FL-MeOH slightly reduced pro-inflammatory cytokines TNF-α and IL-6, attributed to two phytochemicals, naringenin-7-O-α-L-arabinofuranoside **2** and *D*-(+)-pinitol **5a**. Western blot analysis also showed that adipocytes treated with MeOH extracts had higher GLUT-4 expression levels than untreated adipocytes. Overall, *A. saligna* extracts and their isolated compounds demonstrated anti-lipogenesis activity during 3T3-L1 cell differentiation, modulation of transcriptional levels of adiponectin, PGC-1α, and mtTFA, reducing TNF-α and IL-6 mRNA levels, promoting mitochondrial biogenesis, and enhancing GLUT-4 expression.

## 1. Introduction

**Figure 1:**
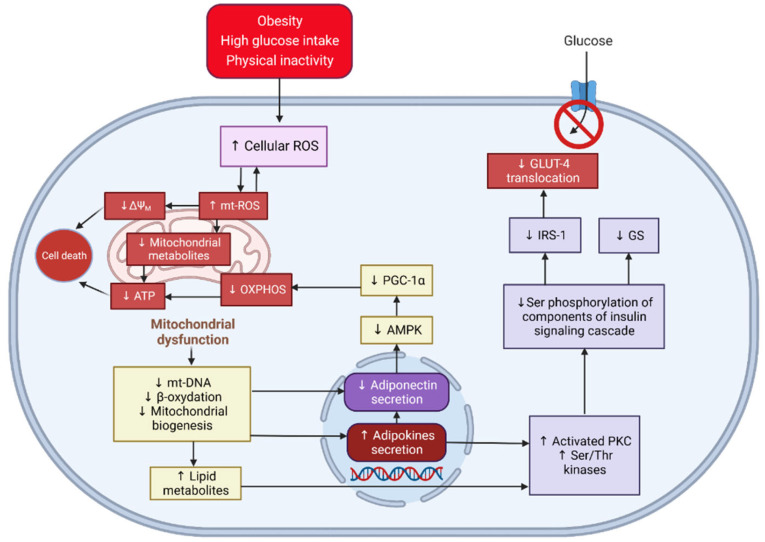
A proposed link between mitochondrial dysfunction and insulin resistance in an adipocyte model. The increased intracellular ROS triggered by obesity and metabolic challenges (e.g., excess nutrient intake) leads to mt-ROS overproduction, exacerbating the total ROS generation and decreasing MMP and ATP production [13]. The impaired mitochondria reflected by the collapse of mt-DNA content and depletion of mitochondrial biogenesis alter adiponectin level, resulting in decreased OXPHOS caused by reduced activation of AMPK and PGC-1α as its regulator [14]. In addition, the compromised mitochondria function causes the accumulation of lipid metabolites because of the reduced β-oxidation and the increase in pro-inflammatory cytokines leading to the induction of PKC isoforms and Ser/Thr kinases [15,16]. Consequently, the inhibition of the insulin signalling cascade, including the disruption of GLUT-4 transduction, leads to the defect of the insulin-dependent glucose uptake pathway. ROS: reactive oxygen species; mt-ROS: mitochondrial reactive oxygen species; MMP: mitochondrial membrane potential; ATP: adenosine 5′-triphosphate; mt-DNA: mitochondrial DNA; OXPHOS: oxidative phosphorylation; AMPK: adenosine 5′-monophosphate-activated protein kinase; PGC-1α: peroxisome proliferator-activated response-γ coactivator-1α; PKC: protein kinase C.

Type 2 diabetes (T2D) is often associated with obesity [1,2,3,4]. In the study by Gao et al. [5], alteration in the mitochondria was observed in 3T3-L1 adipocytes (Figure 1) and in an in vitro model of white adipose tissue. The changes included the overproduction of mitochondrial reactive oxygen species (mt-ROS) and a decrease in mitochondrial membrane potential (MMP). Such changes were linked to oxidative stress and reduced cellular glucose uptake. However, our previous study showed that treatment with Australian *Acacia saligna* extracts and isolated compounds (as listed in Table 1) [6] improved cellular glucose uptake by activating adenosine 5′-monophosphate-activated protein kinase (AMPK) phosphorylation. This treatment also helped restore mitochondrial homeostasis by reducing mt-ROS and improving MMP. However, the biological mechanism for this protection of mitochondria remains unclear. Therefore, this study was conducted to determine the possible mechanism involved in the positive effects of *A. saligna* treatment.

Studies on mitochondrial dysfunction in cell lines [5], mice models [7], and T2D patients [8,9,10] found that levels of peroxisome proliferator-activated receptor-γ coactivators-1 (PGCs-1) were lower than healthy controls. This reduction in PGC-1α can impact mitochondrial transcription factor A (mtTFA), which is essential in regulating the transcription of mitochondrial genes and mitochondrial DNA replication in adipocytes. AMPK has been confirmed as the key regulator of PGCs activation and expression [11]. Furthermore, the activation of PGC-1α requires adiponectin-induced AMPK activation [12]. As *A. saligna* treatment can increase the expression of AMPK, we hypothesised that *A. saligna* extracts and isolated compounds could enhance the expression of adiponectin, PGC-1α, and mtTFA. Therefore, this study evaluated the effects of methanolic extracts and their constituents on transcriptional levels of these vital genes involved in mitochondrial biogenesis using quantitative reverse transcription–polymerase chain reaction (RT-qPCR) analysis.

**Table 1:** Isolated compounds from the methanolic flower (FL-MeOH), leaf (LF-MeOH), and bark (BK-MeOH) extracts of *A. saligna* [6].

Compounds	Extract/Amount (%*w*/*w* Extract)	Compound	Extract/Amount (%*w*/*w* Extract)
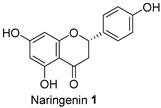	FL-MeOH/1.75	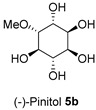	LF-MeOH/8
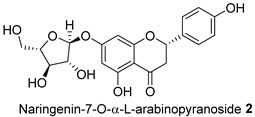	FL-MeOH/2.58	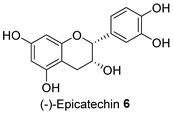	LF-MeOH and BK-MeOH/0.9 ^b^, 2.53 ^c^
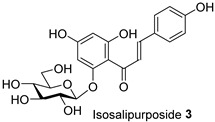	FL/MeOH/1.52	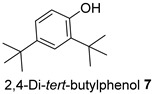	LF-MeOH/1
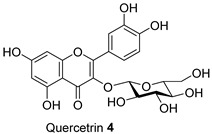	FL-MeOH and LF-MeOH/4.13 ^a^, 2.68 ^b^	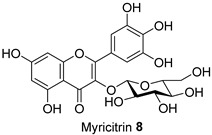	LF-MeOH/5
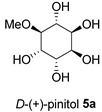	FL-MeOH and BK-MeOH/2.5 ^a^, 17.83 ^c^	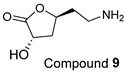	LF-MeOH/5

^a^ from FL-MeOH, ^b^ from LF-MeOH; ^c^ from BK-MeOH.

Interestingly, when mitochondrial regulators are downregulated, there is a significant decrease in phosphorylated Akt and glucose transporter 4 (GLUT-4) levels, along with an increase in the production of pro-inflammatory markers that impede glucose uptake in adipocytes [15,17]. Additionally, along with GLUT-4 oxidation and carbonylation, the excessive triglyceride deposits in adipocytes can promote the pro-inflammatory cytokines, such as tumour necrosis factor α (TNF-α) and interleukin-6 (IL-6), resulting in decreased GLUT-4 expression [18]. The levels of cytokines increase proportionally to adiposity and insulin resistance [19]. Our previous study showed pronounced glucose uptake modulation [20], which could be attributed to improved mitochondrial function that protects GLUT-4. However, the study has not provided empirical information yet to support the hypothesis. Thus, this study also assesses the protein level of GLUT-4 in the adipocytes after treatment with *A. saligna* by conducting an immunoblot analysis. To further ensure the anti-inflammatory beneficial effect of the extract and isolated compounds, the TNF-α and IL-6 mRNA levels were also measured by RT-qPCR.

## 2. Results and Discussion

### 2.1. Lipid Accumulation in 3T3-L1 Adipocytes Treated with Extracts and Isolated Compounds

**Figure 2:**
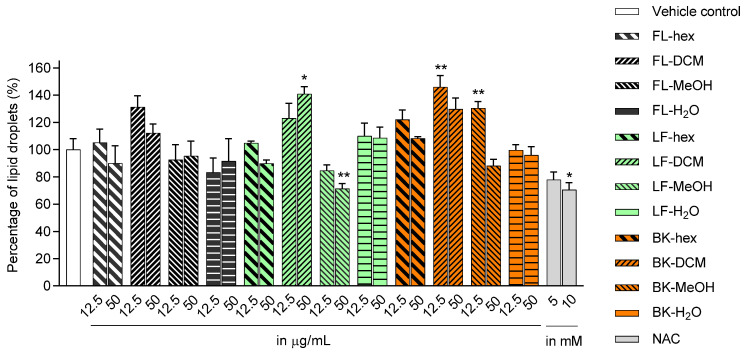
Lipid content from ORO staining assay on the 3T3-L1 adipocytes treated with extracts during the progress of cell differentiation. FL-hex: hexane extract of flowers, FL-DCM: dichloromethane extract of flowers, FL-MeOH: methanol extract of flowers, FL-H_2_O: aqueous extract of flowers, LF-hex: hexane extract of leaves, LF-DCM: dichloromethane extract of leaves, LF-MeOH: methanol extract of leaves, LF-H_2_O: aqueous extract of leaves, BK-hex: hexane extract of bark, BK-DCM: dichloromethane extract of bark, BK-MeOH: methanol extract of bark, BK-H_2_O: aqueous extract of bark, and NAC = *N*-acetyl cysteine. Data in mean ± SEM, * *p* = 0.02 of LF-DCM 50 µg/mL and NAC 10 µM; ** *p* = 0.006 of BK-DCM 12.5 µg/mL and BK-MeOH 12.5 µg/mL; ** *p* = 0.009 of LF-MeOH 50 µg/mL, vs. vehicle control (*n* = 3, One-way ANOVA, Tukey’s post hoc).

Based on the data presented in Figure 2, there were varying amounts of lipid droplets in the adipocytes treated with extracts compared to the control group. The LF-MeOH-treated adipocytes at 50 µg/mL showed a significant decrease in lipid droplets by 28.68% compared to the control, indicating antiadipogenic activity. A similar effect was observed in the positive control treatment with *N*-acetyl cysteine (NAC) at 10 mM for 29.55%. A slight decrease in lipid droplets was observed from treatment with hexane leaf extract (LF-hex) 50 µg/mL, BK-MeOH 50 µg/mL, LF-MeOH 12.5 µg/mL, FL-H_2_O 12.5 µg/mL, and NAC 5 mM by 10.18, 11.9, 15.35, 16.81, and 22.14%, respectively. Conversely, 12.5 µg/mL of BK-MeOH, 50 µg/mL of dichloromethane leaf extract (LF-DCM), and 12.5 µg/mL of dichloromethane bark extract (BK-DCM) significantly increased lipid droplets by 30.4%, 40.9%, and 45.9%, respectively.

**Figure 3:**
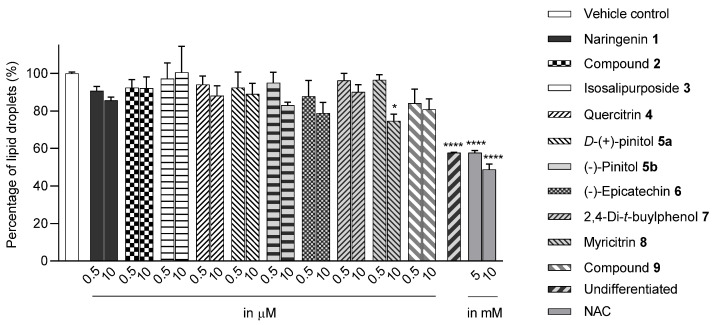
Estimated lipid content from ORO staining assay on the 3T3-L1 adipocytes treated with isolated compounds during differentiation. NAC = *N*-acetyl cysteine. Data in mean ± SEM, * *p* = 0.03 of compound **8** 10 µM; **** *p* = 0.00008 of undifferentiated group and NAC, vs. vehicle control (*n* = 3, One-way ANOVA, Tukey’s post hoc).

The Oil Red O (ORO) staining assay was then used to analyse the effect of isolated compounds from methanolic extracts on lipid content in 3T3-L1 adipocytes. Our results showed that most isolated compounds had anti-lipogenic activity during the differentiation process of 3T3-L1 cells, as shown in Figure 3. The compounds tested induced greater reductions in lipid droplet percentages at 10 μM compared to the vehicle control. At 10 μM, a slight reduction was observed for 2,4-di-*t*-buylphenol **7** (9.86%), *D*-(+)-pinitol **5a** (10.88%), quercitrin **4** (11.88%), naringenin **1** (14.41%), and (−)-pinitol **5b** (16.92%). Furthermore, the lactone derivative **9** and (−)-epicatechin **6** showed moderate reductions in lipid droplets estimated at 19.13 and 21.15%, respectively. Myricitrin **8** significantly reduced lipid accumulation by 25.28%, seven times higher than that at 0.5 μM. This activity reflects the potent inhibition in lipid production by its corresponding LF-MeOH extracts. However, naringenin-*7*-*O*-*α*-*L*-arabinofuranoside **2** did not alter the lipid droplet content at 0.5 and 10 μM, while isosalipurposide **3** did not reduce lipid accumulation at 10 μM.

### 2.2. Expression of mRNAs Related to the Mitochondrial Biogenesis of Adipocytes

Our initial study has revealed that active extracts and compounds found in *A. saligna* can enhance glucose uptake by activating AMPK-α in adipocytes and improving mitochondrial function [20]. Since activation of AMPK is also linked to the biogenesis of mitochondria, we believe that these extracts and phytochemicals may also increase the expression of mtTFA by upregulating PGC-α, which is known to be linked to the biogenesis of mitochondria [12]. To determine the effect of methanolic extracts and isolated compounds on the transcriptional levels of key genes involved in mitochondrial biogenesis, such as adiponectin, PGC-1α, and mtTFA, we conducted a quantitative reverse transcription–polymerase chain reaction (RT-qPCR) study. The expression of target mRNAs, adiponectin, PGC-1α, and mtTFA, increased with the treatment of the three methanolic extracts of *A. saligna*. All three MeOH extracts at 12.5 μg/mL slightly increased the expression of adiponectin by approximately 7% compared to the control group. At 50 μg/mL, the expression of adiponectin improved, and the FL-MeOH extract showed a significant increase in expression, which was 28.92% higher than the control.

**Figure 4:**
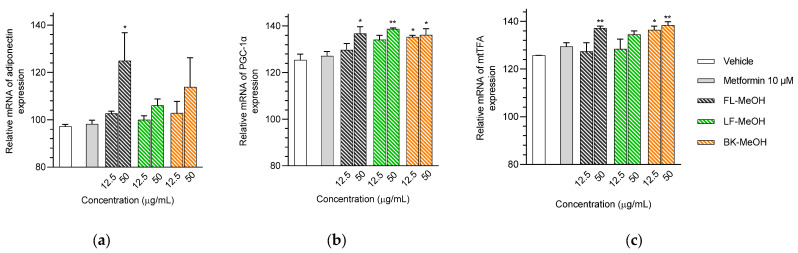
The relative expression of (**a**) mRNA of adiponectin, (**b**) PGC-1α, and (**c**) mtTFA of 3T3-L1 adipocytes treated with MeOH extracts. The gene expression was normalised by the housekeeping gene β-actin. FL-MeOH = methanolic extract of flower, LF-MeOH = methanolic extract of leaf, BK-MeOH = methanolic extract of bark. Data in mean ± SEM; * *p* = 0.01 of FL-MeOH and BK-MeOH 50 µg/mL in experiment (**b**) and BK-MeOH 12.5 µg/mL in experiment (**c**); * *p* = 0.02 of FL-MeOH in experiment (**a**); * *p* = 0.03 of BK-MeOH 12.5 µg/mL in experiment (**b**); ** *p* = 0.002 of LF-MeOH in experiment (**b**); ** *p* = 0.003 of BK-MeOH 50 µg/mL in experiment (**c**); ** *p* = 0.006 of FL-MeOH in experiment (**c**), vs. vehicle control (*n* = 3, One-way ANOVA, Tukey’s post hoc).

Furthermore, at 12.5 μg/mL, BK-MeOH showed small increases in the expression of PGC-1α and mtTFA by 9.9 and 10.7%, respectively. Moreover, the expression of PGC-1α was slightly more upregulated when treating FL-, LF-, and BK-MeOH at 50 μg/mL by 11.4, 13.4, and 10.7%, respectively. On the other hand, only adipocytes treated with FL- and BK-MeOH extracts at 50 μg/mL showed noticeable increases in mtTFA expression by 11.5 and 12.6%, respectively. Figure 4 and Table S4 demonstrate that all three extracts consistently affected target gene expression. Our results align with our previous study [20], showing a twofold increase in mitochondrial membrane potential (MMP) from the FL-MeOH treatment at 50 μg/mL. These findings reinforce the beneficial impact of methanolic extracts on adipocyte mitochondrial biogenesis, as evidenced by the reduction in mt-ROS and enhancement of MMP status. This improvement, therefore, was brought about by the transcriptional expression of adiponectin, PGC-1α, and mtTFA.

**Figure 5:**
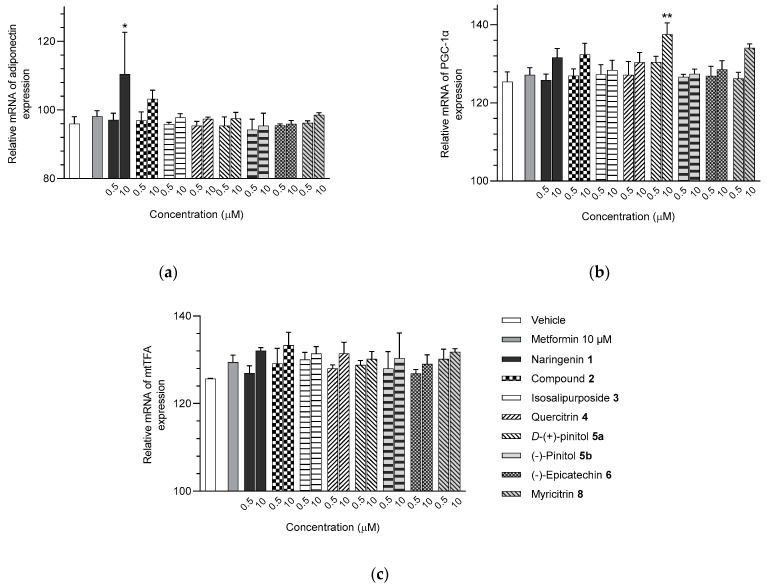
mRNA expression of (**a**) adiponectin, (**b**) PGC-1α, and (**c**) mtTFA in 3T3-L1 adipocytes treated with isolated compounds. The gene expression was normalised by the housekeeping gene β-actin. Data were in mean ± SEM; * *p* = 0.02 of compound **1** 10 µM in experiment (**a**); ** *p* = 0.002 of compound **5a** 10 µM in the experiment (**b**), vs. vehicle control (*n* = 3, One-way ANOVA, Tukey’s post hoc).

At a concentration of 0.5 μM, all isolated compounds caused a slight increase in transcriptional levels of adiponectin, PGC-1α, and mtTFA mRNA, as shown in Figure 5 and Table S5. However, when incubated at 10 μM, naringenin **1** resulted in a 14.42% increase in adiponectin expression, while (+)-pinitol **5a** led to a 12.1% increase in PGC-1α expression.

The findings demonstrate a correlation between the observed increases in MMP by 1.67- and 2-fold when treated with naringenin **1** and *D*-(+)-pinitol **5a** in the adipocytes [20]. Notably, naringenin **1** and *D*-(+)-pinitol **5a** were found in FL-MeOH extract with percentages (*w*/*w*) of 1.75 and 2.5%, respectively [20]. The presence of these two active compounds suggests the noticeable effect of FL-MeOH extract on the increase in gene expression. Previous studies have shown that naringenin **1** can modulate mRNA expression of adiponectin and PGC-1α in human white adipocyte cultures, leading to increased energy expenditure and insulin sensitivity [21]. Additionally, in obese mice, *D*-(+)-pinitol **5a** stimulated PGC-1α mRNA expression by upregulating the cAMP response element-binding protein (CREB) [22].

### 2.3. Expression of mRNAs of Inflammatory Markers of Adipocytes

Our previous study found that methanolic extracts from *A. saligna* and isolated compounds effectively reduced cellular ROS and mt-ROS levels in 3T3-L1 adipocytes [20]. This inhibition of mt-ROS production can potentially prevent the inflammatory response that leads to impaired glucose homeostasis [16]. Cytokine levels are directly proportional to adiposity and insulin resistance [18], and thus, the levels of TNF-α and IL-6 mRNA were measured to assess the inhibitory effects of the extracts and isolated compounds on inflammatory markers. This helps explain the mechanism of reduced mt-ROS levels and enhanced glucose uptake in 3T3-L1 adipocytes.

**Figure 6:**
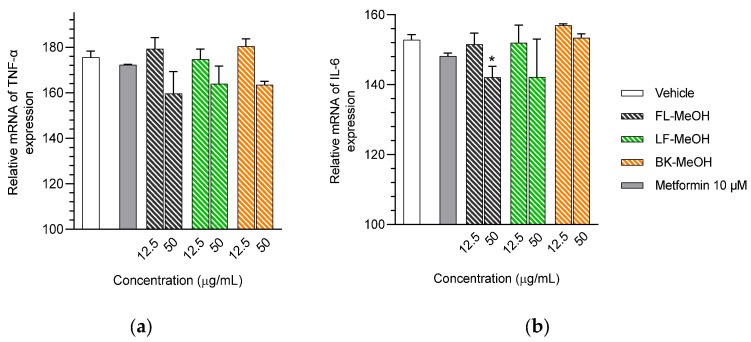
mRNA expression of (**a**) TNF-α and (**b**) IL-6 in 3T3-L1 adipocytes treated with MeOH extracts. The gene expression was normalised by the housekeeping gene β-actin. FL-MeOH = methanolic extract of flower, LF-MeOH = methanolic extract of leaf, BK-MeOH = methanolic extract of bark. Data were in mean ± SEM; * *p* = 0.04 of FL-MeOH 50 µg/mL in the experiment (**b**), vs. vehicle control (*n* = 3, One-way ANOVA, Tukey’s post hoc).

The transcriptional levels of TNF-α and IL-6 were altered in the *A. saligna* MeOH-extract-treated adipocytes compared to the vehicle control group (Figure 6 and Supplementary Table S4). The FL-MeOH-treated adipocytes at 50 μg/mL showed slight decreases in TNF-α and IL-6 expression by approximately 15.9% and 10.5%, respectively. At the same concentration, the LF-MeOH had a slightly lower effect than the methanolic extract of flowers, resulting in 11.6% and 10.4% decreases in TNF-α and IL-6 expression, respectively. On the other hand, the BK-MeOH extract showed a 12% reduction in TNF-α expression and no effect on IL-6 expression. These findings suggest that these extracts have a weak inhibitory effect on pro-inflammatory gene expression in adipocytes.

**Figure 7:**
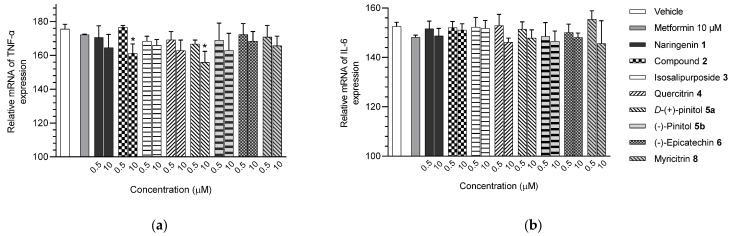
mRNA expression of (**a**) TNF-α and (**b**) IL-6 in 3T3-L1 adipocytes treated with isolated compounds. The gene expression was normalised by the housekeeping β-actin. Data were in mean ± SEM; * *p* = 0.04 of compound **2** 10 µM in the experiment (**a**); * *p* = 0.02 of compound **5a** 10 µM in the experiment (**a**), vs. vehicle control (*n* = 3, One-way ANOVA, Tukey’s post hoc).

The isolated compounds reduced the mRNA expression of two pro-inflammatory adipokines, as shown in Figure 7 and Supplementary Table S5. When treated with naringenin-7-*O*-*α*-*L*-arabinofuranoside **2** and *D*-(+)-pinitol **5a** at 10 μM, there were slight decreases in TNF-α expression by 14.2 and 19.6%, respectively. Additionally, treatment with naringenin **1** and quercitrin **4** at the same dose decreased TNF-α expression by 11 and 12.7%, respectively. However, there was only a minimal inhibitory effect on the expression of IL-6 mRNA when treated with naringenin **1**, quercitrin **4**, *D*-(+)-pinitol **5a**, and naringenin-7-*O*-α-*L*-arabinofuranoside **2** at the same concentration. These results suggested that FL-MeOH inhibited TNF-α expression mainly through naringenin **1**, quercitrin **4**, and compounds **2** and **5a**. Inhibition of IL-6 expression may be due to other unisolated compounds in the extract. Our findings suggest that extracts and phytoconstituents from *A. saligna* can potentially protect adipocyte mitochondria against the harmful effects of pro-inflammatory cytokines, such as TNF-α and IL-6, which have been known to hamper the adiponectin pathway and mitochondrial biogenesis [23,24].

### 2.4. Expression of GLUT-4 of 3T3-L1 Adipocytes Treated with MeOH Extracts

**Figure 8:**
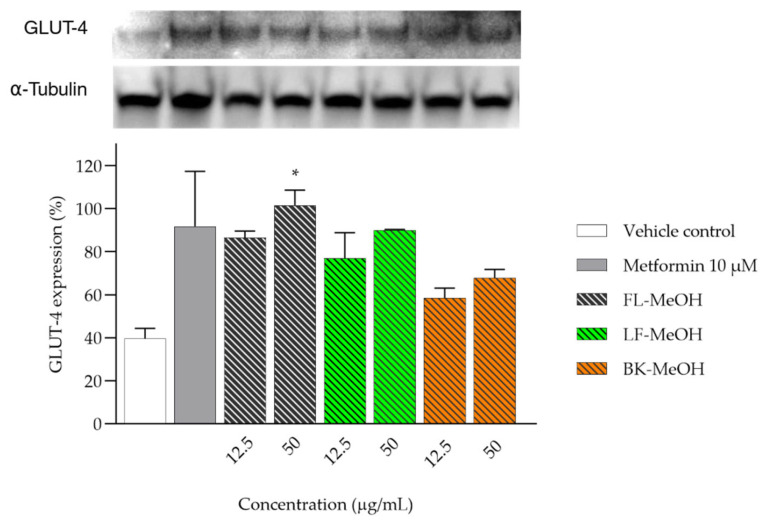
The relative expression of GLUT-4 protein of 3T3-L1 adipocytes treated with MeOH extracts. The expression was normalised by the housekeeping α-tubulin. Data were in mean ± SEM; * *p* = 0.011 of FL-MeOH 50 µg/mL, vs. vehicle control (*n* = 2, One-way ANOVA, Tukey’s post hoc).

Our prior research demonstrated the potential of methanolic extracts and isolated compounds to improve glucose uptake in 3T3-L1 adipocytes [20]. We hypothesised that this effect was attained by activating AMPK and increasing GLUT-4. To validate this pathway, we examined the expression of GLUT-4 in adipocytes. The methanolic extracts of *A. saligna* were found to increase the level of GLUT-4 protein in adipocytes after treatment. This finding is supported by Figure 8 and Supplementary Table S3. The increases in GLUT-4 at a concentration of 12.5 µg/mL were 46.76%, 37.27%, and 18.84% for FL-, LF-, and BK-MeOH, respectively, in comparison to the vehicle control.

Additionally, at a concentration of 50 µg/mL, FL-MeOH treatment significantly increased the GLUT-4 expression by 61.9%, while this effect was weaker for LF- and BK-MeOH treatments, which increased the protein expression by 50.4% and 28.2%, respectively. In other words, treatment with FL-MeOH extract produced the best effect on the increased expression of GLUT-4. This finding supports our earlier report that the adipocytes exposed to FL-MeOH extract had significantly improved glucose uptake by 41.5% and 85.3% at 12.5 and 50 µg/mL, respectively [20]. The study demonstrated a connection between the higher expression of GLUT-4 and the enhanced absorption of glucose in adipocytes. Though the Western blot analysis was repeated twice, additional research with more replications is necessary to establish the accuracy of this initial observation. We previously found that all methanolic extracts could inhibit free radicals [6] by reducing cellular ROS and mt-ROS levels in 3T3-L1 adipocytes [20]. The FL-MeOH extract was particularly effective, with its ability to reduce cellular ROS and mt-ROS by 6.48% to 32%, respectively, in a dose-dependent manner [20]. This reduction in ROS levels has been shown to protect the function of glucose transporters. For example, in H9c2 cells, glucose-induced oxidative stress caused a decrease in GLUT-4 mRNA expression. However, treatment with Trolox restored the expression of the glucose transporter in a concentration- and time-dependent manner [25]. Similarly, in another study, high glucose treatment in HepG2 cells caused a decrease in GLUT-4 protein, which was preserved after incubation with flavonoids such as apigenin and luteolin, which are also natural ROS inhibitors [26].

FL-MeOH showed consistent effects on mitochondrial regulators, pro-inflammatory markers, and glucose transporters. Specifically, when treated with 50 µg/mL of FL-MeOH, there were significant increases in adiponectin, PGC-1α, and mtTFA levels (around one-third, one-tenth, and one-tenth increases, respectively), as well as GLUT-4 expression (three-fifths higher than the vehicle control). Additionally, FL-MeOH treatment resulted in a ~10% reduction in TNF-α and IL-6 mRNA levels. These findings are consistent with previous studies showing that obese mice with increased mitochondrial regulators [27] and reduced cytokines [28] have higher levels of GLUT-4 protein compared to control groups. According to the findings, extracts from *A. saligna* may positively impact mitochondrial homeostasis in adipocytes. This occurred in synergism with the restoration of the GLUT-4 level. Mitochondrial dysfunction can worsen cellular ROS production, inflammatory response, and carbonylation of cellular components [29]. Activating the mitochondrial key regulators with *A. saligna* methanolic extracts can help maintain the adipocytic redox balance. This is shown by a reduction in mt-ROS and MMP values [20], which help preserve GLUT-4 protein from the damaging effects of excessive ROS.

## 3. Materials and Methods

### 3.1. Materials

Unless otherwise expressed, all chemicals were purchased from Sigma-Aldrich (St. Louis, MO, USA). The 3T3-L1 murine cell lines were supplied by American Type Tissue Culture/ATCC (Manassas, VA, USA). The following reagents were purchased from Sigma-Aldrich (St. Louis, MO, USA): Dulbecco’s modified Eagle’s medium high glucose (DMEM), bovine calf serum (BCS), penicillin, streptomycin glutamine (PSG), foetal bovine serum (FBS), rosiglitazone, dexamethasone, 3-isobutyl-1-methylxanthine (IBMX), insulin, phosphate-buffered saline (PBS), trypsin-EDTA solution 0.25%, bovine serum albumin (BSA), metformin, and dimethylsulfoxide (DMSO). Chemicals for cell-based studies were Oil Red O (ORO), formaldehyde solution 10%, and isopropanol.

The following materials for Western blot analysis were purchased from Merck (Darmstadt, Germany): skim milk powder, glycine, hydrochloric acid, sodium dodecyl sulphate (SDS), tris, ammonium peroxide sulphate, sodium hydroxide, acrylamide/bis-acrylamide solution 30%, and tetramethylethylenediamine. The antibodies were from Cell Signalling Technology (Danvers, MA, USA), including anti-rabbit α-tubulin primary antibody and anti-rabbit IgG horseradish peroxidase-linked secondary antibody. The anti-rabbit glucose transporter 4 (GLUT-4, phospho S488) antibody was from Abcam (Waltham, Boston, MA, USA). The following chemicals were purchased from Bio-Rad (Hercules, CA, USA), including the Laemmli sample buffer and all blue protein standards. The other materials were supplied by Thermo-Fisher Scientific (Eugene, OR, USA), including 1.0 mm empty mini gel cassettes, iBlot 2 polyvinylidene fluoride (PVDF) transfer mini stacks, radioimmunoprecipitation assay (RIPA) buffer, enhanced chemiluminescence (ECL) substrate, Tween-20, protease and phosphatase inhibitor, and bicinchoninic acid (BCA) protein assay kits.

The following materials (Table 2) for the RT-qPCR experiments were supplied by Promega (Madison, WI, USA): nuclease-free water, dNTP mix (10 mM), reverse transcription kits of cDNA first-strand synthesis, and random primers. The following target primers (TaqMan™ GeneExpressionAssay (FAM)) were purchased from Thermo-Fisher Scientific (Waltham, MA, USA).

The other materials were a Trizol reagent for RNA isolation (Sigma-Aldrich, St. Louis, MO, USA), a 96-well PCR plate and adhesive seals (Bio-Rad, Hercules, CA, USA), solvents including isopropanol, chloroform, and ethanol (Point of Care Diagnostics, North Rocks, NSW, Australia), and a master mix for RT-PCR (Meridian Bioscience, Cincinnati, OH, USA).

### 3.2. Differentiation of 3T3-L1 Cells

The 3T3-L1 preadipocytes (70–80% confluent from a culture flask) were grown in a 96-well microtiter plate (3 × 10^3^ cells/well in 100 µL final volume of basal medium 1 (M1 = 90% DMEM, 9% BCS, and 1% PSG)) and incubated for 48 h in a humid condition (37 °C and 5% CO_2_) for adherence of the cells. After 48 h, the old M1 was replaced with new M1, and the cells were incubated for another 48 h (day −2 to 0) to get 100% confluent. The M1 was replaced by an identical volume of M2 (9% FBS, 1% PSG, and 90% DMEM containing rosiglitazone 2 µM, dexamethasone 2.2 mM, IBMX 500 mM, and insulin 4 mg/mL), followed by incubation for 48 h (day 0 to 2). After incubation and M2 removal at day 2 of differentiation, new M3 (90% DMEM, 9% FBS, 1% PSG, and insulin) were added, followed by incubation to day 6 with medium replacement every 48 h. On day 6, M3 was replaced by M4 (90% DMEM, 9% FBS, and 1% PSG), followed by another 48 h incubation.

### 3.3. Estimation of Lipid Accumulation Using Oil Red O (ORO) Assay

This assay protocol was adapted from Kraus et al. [30]. On the day of induction of differentiation (day 0), the confluent preadipocytes were exposed to the differentiation medium of induction in the presence of extracts (12.5 and 50 µg/mL) or isolated compounds (0.5 and 10 µM) followed by 48 h incubation. The cells were fed with fresh basal media containing insulin in the presence of the extracts every other 2 days until day 8 of the induction. On the day of staining (day 8), the treated cells and control were washed with PBS and then fixed with 10% formalin solution (dissolved in PBS, 100 µL/well) for 30 min at room temperature. The cells were washed with 60% isopropanol and then stained with 60% Oil Red O (0.7 g of ORO/200 mL of isopropanol, 100 µL/well) for 1 h at room temperature. After liquid removal, the stained cells were washed with water and eluted with 100% isopropanol. The plate was incubated for 10 min at room temperature with a shaker. The absorbance was measured at 510 nm through a microplate reader. The lipid level was expressed by:

$$Fold\;change\;of\;adipogenesis=\frac{\mathrm{Absorbance}\;\mathrm{of}\;\mathrm{treated}\;\mathrm{adipocytes}}{Absorbance\;of\;control}\times 100\%$$

### 3.4. Quantitative Reverse Transcription–Polymerase Chain Reaction (RT-qPCR)

Total RNA was extracted using Trizol reagent, followed by precipitation with isopropyl alcohol, three times washing with 75% EtOH in nuclease-free water, solvent evaporation, and dissolving in nuclease-free water. The concentration of RNA was quantified using a Nanodrop 2000 spectrometer (Thermo-Fisher Scientific, Waltham, MA, USA). The first-strand cDNA was synthesised from 1 µg of the isolated RNA according to the protocol of the manufacturer. The reaction was conducted in a Bio-Rad T100 Thermal Cycler (Hercules, CA, USA). The concentration of cDNA product used as a template for amplification of RT-qPCR was estimated using the nanodrop apparatus. The qPCR was carried out in a Bio-Rad CFX96 Real-Time System (Hercules, CA, USA) following the instructions of the kit. The reaction was conducted in the following stages: polymerase activation (95 °C, 2 min), denaturation (95 °C, 10 s), and the annealing step (60 °C, 30 s) for 50 cycles.

### 3.5. Western Blot Analysis

The Western blot protocol was adapted with modifications from Montt-Guevara et al. [31]. After treatment, the medium of adipocytes was discarded, followed by washing out with pre-chilled PBS, then incubation with RIPA containing protease and phosphatase inhibitor cocktail buffer for 30 min, and moved into microtubes before being centrifuged at 12,000 rpm for 20 min at 4 °C. Subsequently, the supernatant was collected into new microtubes, and the protein concentration was quantified using a BCA protein assay kit. Before separation, the protein supernatant of 35 µg was mixed with loading buffer and boiled at 95 °C on a heating block for 10 min. The electrophoresis was carried out by 10% sodium dodecyl sulphate–polyacrylamide gel electrophoresis (SDS-PAGE) gels for 1 h and 30 min using a Mini Gel Tank (Thermo-Fisher Scientific, Waltham, MA, USA). The separated proteins were transferred into PVDF membranes using an iBlot Western blot transfer system (Thermo-Fisher Scientific, Waltham, MA, USA), followed by blocking with 5% skim milk solution in Tris-buffered saline with Tween (TBST) buffer for an hour. After washing three times with TBST (10 min per each at room temperature), the membranes were incubated with the targeted antibodies solution (1:1000) overnight at 4 °C. After washing with TBST three times, the membranes were incubated with the secondary antibody solution (1:10,000) at room temperature for an hour. Following this, they were rewashed three times with TBST. Finally, the protein strips were detected by the ECL reagents and captured by a Chemidoc system (Amersham Image-quant, Marlborough, MA, USA). The protein expression bands were quantified using Image J (National Institute of Health, Bethesda, MD, USA). The experiment was independently replicated twice.

### 3.6. Statistical Analysis

A descriptive analysis using GraphPad Prism 9 (San Diego, CA, USA) was conducted to determine the mean ± standard error mean (SEM) from the three independent experiments (*n* = 3), except the Western blot analysis (*n* = 2). The difference between the two means of each sample and the vehicle control optical densities was calculated using a one-way ANOVA followed by Tukey’s post hoc tests, where *p* < 0.05 was considered significant.

## 4. Conclusions

Treatment with LF-MeOH extract inhibited adipogenesis of 3T3-L1 cells, as demonstrated by the ORO staining assay. LF-MeOH’s effectiveness is reflected in the significant decrease in lipid accumulation caused by (−)-epicatechin **6** and myricitrin **8**. The methanolic extracts and phytochemicals also increased mRNA levels of key regulators of mitochondrial biogenesis, including adiponectin, PGC-1α, and mtTFA. FL-MeOH treatment showed a consistent increase in PGC-1α and mtTFA levels among the three methanolic extracts. The effect of FL-MeOH on PGC-1α and mtTFA expression is supported by the ability of its isolated compounds, namely naringenin **1** and *D*-(+)-pinitol **5a**, to increase the mRNA expression. All three methanolic extracts slightly reduced the expression of TNF-α and IL-6 at around 10%. However, FL-MeOH was more effective at reducing the expression of IL-6 compared to LF-MeOH and BK-MeOH. The results indicate that these extracts have a weak inhibitory effect on adipocyte pro-inflammatory gene expression. Naringenin **1**, compound **2**, quercitrin **4**, and *D*-(+)-pinitol **5a** are the isolated compounds of FL-MeOH. When tested at a concentration of 10 µM, these compounds showed a slight reduction in TNF-α expression but did not affect IL-6 expression. This suggests that these compounds are responsible for inhibiting TNF-α expression. However, other unisolated compounds in the extract may be responsible for inhibiting IL-6 expression by FL-MeOH. These findings align with the immunoblot analysis outcome, showing that treatment with MeOH extracts increased the expression of GLUT-4 protein in the adipocytes. This suggests that *A. saligna* extracts and isolated compounds can reduce lipid accumulation, promote adipocytes’ mitochondria health, and reduce inflammation, resulting in increased GLUT-4 expression.

## Figures and Tables

**Table 2:** List of target Taqman primers used for the RT-qPCR experiments.

No	Primers	Catalogue No.
1	Adiponectin	4331182/assay ID: Mm04933656_m1
2	PGC-1α	4331182/assay ID: Mm01208835_m1
3	mtTFA	4331182/assay ID: Mm00447485_m1
4	TNF-α	4331182/assay ID: Mm00443258_m1
5	IL-6	4331182/assay ID: Mm00446190_m1
6	β-Actin	4331182/assay ID: Mm02619580_g1

## Data Availability

Data are available within the article.

## Supplementary Materials

The following supporting information can be downloaded at https://www.mdpi.com/article/10.3390/molecules28186677/s1: Experimental procedure: Extraction and compound isolation and identification from the flowers, leaves, and bark of *Acacia saligna*; Table S1: Estimated lipid content from adipogenesis assay with ORO staining agent on the 3T3-L1 adipocytes treated with extracts; Table S2: Estimated lipid content from adipogenesis assay with ORO staining agent on the 3T3-L1 adipocytes treated with isolated compounds within the differentiation process (day-0 to day-8); Table S3: Estimated expression of GLUT-4 normalised by α-tubulin of the 3T3-L1 adipocytes treated with extracts; Figure S1: Original membrane images of Western blot analysis for GLUT-4; Figure S2: Original membrane images of Western blot analysis for α-tubulin; Table S4: Quantitative data of the expression of the target mRNA normalised by β-actin from the RT-qPCR of adipocytes treated with MeOH extracts; Table S5: Quantitative data of the expression of the target mRNA normalised by β-actin from the RT-qPCR of adipocytes treated with MeOH-isolated compounds.

## Sample Availability

Samples of the compounds are not available from the authors.
