# Supplementary material for: Preventing Adipogenesis and Preserving Mitochondria and GLUT-4 Functions by Extracts and Isolated Compounds of Australian Acacia saligna
Source: Molecules. 2023 Sep 18;28(18):6677. doi: 10.3390/molecules28186677 (PMC10535536; doi:10.3390/molecules28186677)
Supplement: Supplementary file 1 [file molecules-28-06677-s001.zip › molecules-2589209-supplementary.pdf]

Supplementary information

Preventing adipogenesis and preserving mitochondria and GLUT-4 functions by extracts and isolated compounds of *Acacia saligna*

Anjar P. Asmara [1], Hui Chen[2], and Alison T. Ung [1,*]

[1] School of Mathematical and Physical Sciences, Faculty of Science, University of Technology Sydney, Ultimo, NSW 2007, Australia
[2] School of Life Sciences, Faculty of Science, University of Technology Sydney, Ultimo, NSW 2007, Australia
* Correspondence: alison.ung@uts.edu.au

Table of contents	Page
**Experimental procedure**: Extraction and compound isolation and identification from the flowers, leaves, and bark of *Acacia saligna*	2
**Table S1.** Estimated lipid content from adipogenesis assay with ORO staining agent on the 3T3-L1 adipocytes treated with extracts	2
**Table S2**. Estimated lipid content from adipogenesis assay with ORO staining agent on the 3T3-L1 adipocytes treated with isolated compounds within the differentiation process (day-0 to day-8)	2
**Table S3**. Estimated expression of GLUT-4 normalised by α-tubulin of the 3T3-L1 adipocytes treated with extracts	3
**Figure S1.** Original membrane images of Western blot analysis for GLUT-4	3
**Figure S2.** Original membrane images of Western blot analysis for α-tubulin	4
**Table S4**. Quantitative data of the expression of the target mRNA normalised by β-actin from the RT-qPCR of adipocytes treated with MeOH extracts	5
**Table S5**. Quantitative data of the expression of the target mRNA normalised by β-actin from the RT-qPCR of adipocytes treated with MeOH-isolated compounds	6

1. **Experimental procedure**: Extraction and compound isolation and identification from the flowers, leaves, and bark of *Acacia saligna*

Successive solvent extraction with *n*-hexane, dichloromethane (DCM), methanol (MeOH), and water solvent gave twelve different types of extracts of *A. saligna* [28]. Using a bioassay-guided fractionation, eleven compounds were obtained from FL-, LF-, and BK-MeOH extracts through isolation using column chromatography techniques [28]. Spectroscopic methods using FTIR, 1D and 2D NMR, and HRMS suggested the presence of various classes of compounds, including chalcone, flavanone, flavan-3-ol, flavonol, alkyl phenol, cyclitols, and lactone derivatives. Five compounds were isolated from FL-MeOH, including naringenin **1**, naringenin-*7-O-α-L*-arabinofuranoside **2**, isosalipurposide **3**, quercitrin **4**, and *D*-(+)-pinitol **5a**. Flavonoid derivatives, including quercitrin **4**, (−)-epicatechin **6**, and myricitrin **8** were the most isolated compounds found in the LF-MeOH, followed by 2,4-di-*t*-butylphenol **7**, (−)-pinitol **5b**, and (*3S*,5S**)-3-hydroxy-5-(2-aminoethyl) dihydrofuran-2(3*H*)-one **9**. Isolated phytochemicals BK-MeOH were (−)-epicatechin **6**, *D*-(+)-pinitol **5a**, and sucrose.

2. **Data of the studies**

Table S1. Estimated lipid content during adipogenesis using ORO staining agent on the 3T3-L1 adipocytes treated with extracts.

No	Sample	Fold change of adipogenesis (%)	
		**12.5 μg/mL**	**50 μg/mL**
**1**	Vehicle control	100 ± 8.13	
**2**	FL-hex	105.1 ± 10.08	90.04 ± 12.87
**3**	FL-DCM	131.3 ± 8.38	112.2 ± 6.66
**4**	FL-MeOH	92.45 ± 11.17	95.36 ± 10.84
**5**	FL- H_2O	83.19 ± 10.67	91.46 ± 16.75
**6**	LF-hex	104.8 ± 1.46	89.82 ± 2.54
**7**	LF-DCM	123 ± 10.97	140.9 ± 5.32*
**8**	LF-MeOH	84.65 ± 4.08	71.32 ± 3.77**
**9**	LF- H_2O	110 ± 9.52	108.5 ± 7.98
**10**	BK-hex	122.1 ± 7.01	108.3 ± 1.12
**11**	BK-DCM	145.9 ± 8.56**	129.7 ± 8.21
**12**	BK-MeOH	130.4 ± 4.94**	88.1 ± 4.73
**13**	BK-H_2O	99.56 ± 4.16	95.95 ± 6.30
**14**	NAC 5 mM	77.86 ± 5.77	
**15**	NAC 10 mM	70.45 ± 5.45*	

Data in mean ± SEM; *p = 0.02 of LF-DCM 50 μg/mL and NAC 10 μM; **p = 0.006 of BK-DCM 12.5 μg/mL and BK-MeOH 12.5 μg/mL; **p = 0.009 of LF-MeOH 50 μg/mL, *vs* vehicle control (n = 3, one-way ANOVA, Tukey's post hoc tests).

Table S2. Estimated lipid content during adipogenesis using ORO staining agent on the 3T3-L1 adipocytes treated with isolated compounds within the differentiation process (day-0 to day-8)

No	Sample	Fold change of adipogenesis (%)	
		**0.5 μM**	**10 μM**
**1**	Vehicle control	100 ± 0.81	
**2**	Naringenin **1**	90.69 ± 2.41	85.59 ± 1.77
**3**	Naringenin-*7O-α-L*-arabinofuranoside **2**	92.41 ± 4.34	92.16 ± 5.98
**4**	Isosalipurposide **3**	97.18 ± 8.43	100.5 ± 13.98
**5**	Quercitrin **4**	94.1 ± 4.59	88.12 ± 5.32
**6**	*D*-(+)-pinitol **5a**	92.38 ± 8.41	89.12 ± 5.64
**7**	(−)-Pinitol **5b**	94.99 ± 5.72	83.08 ± 1.58
**8**	(−)-Epicatechin **6**	87.85 ± 8.40	78.85 ± 5.68

No	Sample	Fold change of adipogenesis (%)	
		0.5 µM	10 µM
**9**	2,4-Di-*t*-butylphenol **7**	96.27 ± 3.76	90.14 ± 3.85
**10**	Myricitrin **8**	96.55 ± 2.78	74.72 ± 3.67*
**11**	3-Hydroxy-5-(2-aminoethyl) dihydrofuran-2(3*H*)-one **9**	84.12 ± 7.54	80.87 ± 5.62
**12**	NAC 5 mM	57.69 ± 1.16****	
**13**	NAC 10 mM	48.87 ± 2.86****	
**14**	Undifferentiated	57.70 ± 0.25****	

Data in mean ± SEM; *p = 0.03 of compound **8** 10 µM; ****p = 0.00008 of undifferentiated group and NAC, *vs* vehicle control (n = 3, one-way ANOVA, Tukey's post hoc tests).

Table S3. GLUT-4 level normalised by α-tubulin in 3T3-L1 adipocytes treated with extracts.

No	Sample	GLUT-4 expression (%)	
		12.5 µg/mL	50 µg/mL
**1**	Vehicle control	39.7 ± 4.67	
**2**	Metformin 10 µM	91.82 ± 25.6	
**3**	FL-MeOH	86.46 ± 3.21	101.6 ± 7.00 *
**4**	LF-MeOH	76.97 ± 11.93	90.1 ± 0.34
**5**	BK-MeOH	58.54 ± 4.56	67.9 ± 3.91

Data in mean ± SEM; *p = 0.042 of FL-MeOH 50 µg/mL, *vs* vehicle control (n = 2, one-way ANOVA, Tukey's post hoc tests).

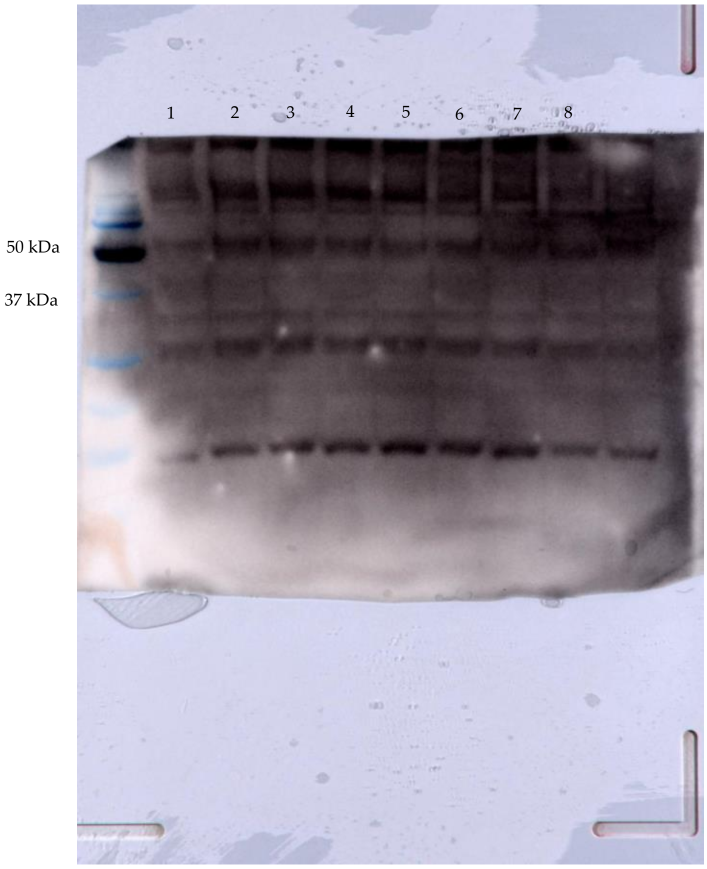

Figure S1. Original membrane images of Western blot analysis of GLUT-4 (lane 1 = vehicle control, 2 = metformin 10 μM, 3 = FL-MeOH 12.5 μg/mL, 4 = FL-MeOH 50 μg/mL, 5 = LF-MeOH 12.5 μg/mL, 6 = LF-MeOH 50 μg/mL, 7 = BK-MeOH 12.5 μg/mL, and 8 = BK-MeOH 50 μg/mL)

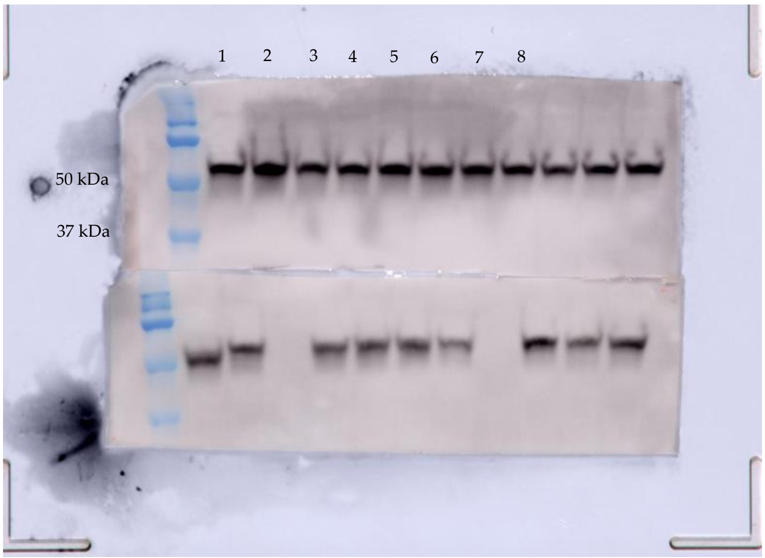

Figure S2. Original membrane images of Western blot analysis of α-tubulin (lane 1 = vehicle control, 2 = metformin 10 μM, 3 = FL-MeOH 12.5 μg/mL, 4 = FL-MeOH 50 μg/mL, 5 = LF-MeOH 12.5 μg/mL, 6 = LF-MeOH 50 μg/mL, 7 = BK-MeOH 12.5 μg/mL, and 8 = BK-MeOH 50 μg/mL).

Table S4. mRNA expression of target genes normalised by β-actin in adipocytes treated with MeOH extracts

Treatment	Concentration	mRNAs related to mitochondrial biogenesis			Inflammatory markers' mRNAs	
		Adiponectin	PGC-1α	mtTFA	TNF-α	IL-6
Vehicle control	-	95.98 ± 2.08	125.4 ± 2.50	125.7 ± 0.08	175.5 ± 2.81	152.6 ± 1.65
Metformin	10 μM	98.15 ± 1.67	127.1 ± 1.9	129.4 ± 1.63	172.2 ± 0.23	148.2 ± 0.83
FL-MeOH	12.5 μg/mL	102.6 ± 1.00	129.7 ± 2.76	127.4 ± 3.58	179.3 ± 4.87	151.5 ± 3.25
	50 μg/mL	124.9 ± 11.87*	136.8 ± 2.91*	137.2 ± 0.81**	159.6 ± 9.72	142.1 ± 3.19*
LF-MeOH	12.5 μg/mL	99.94 ± 1.68	134.1 ± 1.85	128.4 ± 4.16	174.7 ± 4.39	151.9 ± 5.10
	50 μg/mL	106.1 ± 2.72	138.8 ± 0.36**	134.4 ± 1.53	163.9 ± 7.75	142.2 ± 10.89
BK-MeOH	12.5 μg/mL	102.8 ± 4.94	135.3 ± 0.69*	136.4 ± 1.69*	180.3 ± 3.35	156.9 ± 0.45
	50 μg/mL	113.8 ± 12.33	136.1 ± 2.71*	138.3 ± 1.47**	163.5 ± 1.56	153.4 ± 1.22

Data in mean ± SEM; *$p = 0.01$ of FL-MeOH and BK-MeOH 50 μg/mL in PGC-1α experiment and BK-MeOH 12.5 μg/mL in mtTFA experiment; *$p = 0.02$ of FL-MeOH in adiponectin experiment; *$p = 0.03$ of BK-MeOH 12.5 μg/mL in PGC-1α experiment; **$p = 0.002$ of LF-MeOH in PGC-1α experiment; **$p = 0.003$ of BK-MeOH 50 μg/mL in mtTFA experiment; **$p = 0.006$ of FL-MeOH in mtTFA experiment, *vs* vehicle control ($n = 3$, one-way ANOVA, Tukey's post hoc tests).

Table S5. mRNA expression of target genes normalised by β-actin in adipocytes treated with MeOH isolated compounds

Treatment	Concentration (μM)	mRNAs related to mitochondrial biogenesis			Inflammatory markers' mRNAs	
		**Adiponectin**	**PGC-1*α***	**mtTFA**	**TNF-*α***	**IL-6**
Vehicle control	-	95.98 ± 2.08	125.4 ± 2.5	125.7 ± 0.08	175.5 ± 2.81	152.6 ± 1.65
Metformin	10	98.15 ± 1.67	127.1 ± 1.9	129.4 ± 1.63	172.2 ± 0.23	148.2 ± 0.83
Naringenin **1**	0.5	97.07 ± 1.97	125.8 ± 1.52	126.9 ± 1.72	170.6 ± 6.91	151.6 ± 3.16
	10	110.4 ± 12.19*	131.6 ± 2.33	132.1 ± 0.73	164.5 ± 7.90	148.7 ± 3.03
Naringenin-7*O*-*α*-*L*-arabinofuranoside **2**	0.5	96.93 ± 2.52	127 ± 1.71	129.1 ± 3.44	176.6 ± 1.14	152 ± 2.56
	10	103.2 ± 2.56	132.4 ± 2.86	133.3 ± 2.96	161.3 ± 5.45*	151.1 ± 2.52
Isosalipurposide **3**	0.5	95.85 ± 0.55	127.3 ± 2.46	130 ± 1.69	168.4 ± 2.88	152.3 ± 3.95
	10	97.8 ± 1.14	128.4 ± 2.54	131.4 ± 1.61	165.9 ± 3.54	151.7 ± 3.22
Quercitrin **4**	0.5	95.38 ± 1.30	127.1 ± 3.45	128 ± 0.85	169.2 ± 4.88	152.9 ± 4.52
	10	97.38 ± 0.54	130.4 ± 2.52	131.4 ± 2.55	162.8 ± 6.23	146.1 ± 1.68
D-(+)-Pinitol **5a**	0.5	95.4 ± 2.59	130.4 ± 1.51	128.8 ± 1.04	166.7 ± 2.32	151.4 ± 2.98
	10	97.49 ± 1.83	137.5 ± 2.89**	130.2 ± 1.73	155.9 ± 6.45*	147.9 ± 3.29
(–)-Pinitol **5b**	0.5	94.24 ± 3.10	126.6 ± 0.71	128 ± 3.84	169 ± 10.08	148.5 ± 5.62
	10	95.41 ± 3.66	127.4 ± 1.24	130.3 ± 5.79	163 ± 10.05	146.5 ± 4.17
(–)-Epicatechin **6**	0.5	95.42 ± 0.52	126.9 ± 2.46	126.8 ± 0.93	172.3 ± 6.54	150.1 ± 3.41
	10	95.92 ± 1.03	128.5 ± 2.25	129 ± 2.10	168.4 ± 5.74	148.1 ± 1.79
Myricitrin **8**	0.5	96.18 ± 0.69	126.2 ± 1.59	130.2 ± 2.25	170.9 ± 6.85	155.4 ± 3.44
	10	98.52 ± 0.70	134.1 ± 1.02	131.8 ± 0.72	165.6 ± 5.68	145.6 ± 9.24

Data in mean ± SEM; *$p = 0.02$ of compound **1** 10 μM in adiponectin experiment and compound **5a** 10 μM in TNF-*α* experiment; *$p = 0.04$ of compound **2** 10 μM in TNF-*α* experiment; **$p = 0.002$ of compound **5a** 10 μM in PGC-1*α* experiment, *vs* vehicle control ($n = 3$, one-way ANOVA, Tukey's post hoc tests).
